# Classification moléculaire du cancer du sein au Maroc

**Published:** 2012-12-31

**Authors:** Abbass Fouad, Akasbi Yousra, Znati Kaoutar, El Mesbahi Omar, Amarti Afaf, Bennis Sanae

**Affiliations:** 1 Laboratoire des molécules bioactives, Faculté des Sciences et Techniques de Fès, Maroc; 2 Service d'oncologie médicale, CHU Hassan II Fès, Maroc; 3 Service Anatomo-pathologique CHU Hassan II de Fès, Maroc; 4 Laboratoire de biologie des cancers, Faculté de Médecine et de Pharmacie de Fès, Maroc; 5 Unité d'oncogénétique, CHU Hassan II de Fès, Maroc

**Keywords:** Cancer du sein, prévalence, pronostic, groupes moléculaires, biomarqueurs, immunohistochimie, survie, breast cancer, prevalence, prognosis, molecular group, biomarkers, immunohistochemistry, survival

## Abstract

**Introduction:**

La classification moléculaire des cancers du sein basée sur l'expression génique puis sur le profil protéique a permis de distinguer cinq groupes moléculaires: luminal A, luminal B, Her2/neu, basal-like et non-classées. L'objectif de cette étude réalisée au CHU Hassan II de Fès est de classer 335 cancers du sein infiltrant en groupes moléculaires, puis de les corréler avec les caractéristiques clinicopathologiques.

**Méthodes:**

Etude rétrospective étalée sur 45 mois, comportant 335 patientes colligées au CHU pour le diagnostic et le suivi. Les tumeurs sont analysées histologiquement et classées après une étude immunohistochimique en groupes: luminal A, luminal B, Her2+, basal-like et non-classées.

**Résultats:**

54.3% des tumeurs sont du groupe luminal A, 16% luminal B, 11.3% Her2+, 11.3% basal-like et 7% non-classées. Le groupe luminal A renferme le plus faible taux de grade III, d'emboles vasculaires ainsi que de métastases; alors que le groupe des non-classées et basal-like représentent un taux élevé de grade III, une faible proportion d'emboles vasculaires et d'envahissement ganglionnaire. Ces facteurs sont significativement élevés dans les groupes luminal B et Her2+ avec un taux de survie globale de 78% et 76% respectivement. Dans le groupe luminal A, la survie globale des patientes est élevée (87%) alors qu'elle n'est que de 49% dans le groupe des triples négatifs (basal-like et non-classés).

**Conclusion:**

Le groupe luminal B est différent du luminal A et il est de pronostic péjoratif vis à vis du groupe Her2+. Les caractéristiques clinicopathologiques concordent avec le profil moléculaire donc devraient être pris en considération comme facteurs pronostiques.

## Introduction

Le cancer du sein est le premier cancer de la femme. Il est responsable de 11000 décès par an et il reste jusqu’à l'heure actuelle un problème de santé publique particulièrement pour la femme jeune, moins de 35 ans, où il est agressif [1]. Cependant, la stratégie thérapeutique de nos jours s'est améliorée grâce à l'utilisation des traitements adjuvants qui ont permis de réduire au maximum le risque de récidives [2] et le taux de mortalité ainsi la survie globale à 10 ans est augmentée (atteint 70%) [3]. Ces nouveaux traitements ont également augmenté la difficulté de déterminer avec précision le traitement adéquat qui changera le pronostic. Pour remédier à ce problème, Perou-Sorlie et d'autres auteurs [4–8] ont analysé l'expression génique des tumeurs mammaires par microarray et ont pu mettre en évidence cinq groupes moléculaires: luminal A, luminal B, basal-like, Her2/neu (surexpression du Her2) et le groupe normal-like. Ce dernier fut écarté car il est artefactuel et correspond à des tumeurs contaminées par un tissu mammaire sain [8]. Cependant, les analyses par microarray n'est pas toujours possible vu leur coût et les difficultés techniques pour réaliser ces analyses. Pour résoudre ce problème plusieurs auteurs ont démontré que l'immunohistochimie peut servir de surrogate au microarray pour définir les sous types moléculaires de la classification intrinsèque [9–13]. Ainsi, Carey [9], Cheang [10] et d'autres auteurs [11–13] ont ensuite reproduit par immunohistochimie l'expression protéique des tumeurs mammaires, en se basant sur l'expression des récepteurs oestrogéniques (RE), progestéroniques (RP), facteur de croissance Her2, Ki-67 et d'autres biomarqueurs tels les cytokératines de haut et de bas poids moléculaires (CK8 /18, CK5/6 etc..). Ces auteurs ont ainsi mis en évidence le groupe moléculaire des non-classés (tumeurs négatives à tous les biomarqueurs) à côté des groupes luminal A, luminal B, Her2/neu et basal-like. Le groupe des tumeurs luminal B se distingue des tumeurs luminal A par une forte prolifération mitotique détectée par l'anticorps Ki-67 [10].

L'objectif de cette étude, réalisée pour la première fois au Maroc, est de classer les carcinomes mammaires infiltrants colligés au CHU Hassan II de Fès selon le profil protéique des tumeurs en groupes moléculaires, puis de corréler ces derniers aux caractéristiques clinico-pathologiques des tumeurs.

## Méthodes

Il s'agit d'une étude rétrospective étalée sur 45 mois (à partir de janvier 2007), comportant 335 patientes atteintes d'un carcinome infiltrant du sein et recrutées au CHU Hassan II de Fès au Maroc pour le diagnostic et le suivi. L’étude est réalisée sur des prélèvements biopsiques et des pièces opératoires fixés dans le formol à 10% et inclus en paraffine. Les tumeurs de ces patientes sont analysées histologiquement et gradées selon le système de Scarff, Bloom et Richardson (SBR) [14, 15]. L'analyse intéresse les variables suivantes: âge, taille tumorale, grade histologique SBR, envahissement ganglionnaire, métastases à distance, statut RE/RP/Her2.

### Etude immunohistochimique (IHC)

Les tumeurs sont classées en cinq groupes moléculaires en se basant sur les résultats immunohistochimiques: luminal A (ER+ et/ou PR+, Her2-, CK8/18+ et Ki-67 ‘ 14%), luminal B (ER+ et/ou PR+, Her2+, CK8/18+ et Ki-67 > 14%), Her2+ (ER-, PR-, Her2+), basal-like (ER-, PR-, Her2-, CK 5/6+ et/ou EGFR+) et non-classés (ER, PR-, Her2-, CK 5/6- et EGFR-).

La technique est réalisée avec l'appareil Ventana Benchmark^®^ XT en mode semi-automatique et les anticorps utilisés sont dilués au ½: RE (clone ER1D5); RP (clone PR10A9); CK5/6 (clone D5/16B4); CK8/18 (clone 5D3); EGFR (clone 3C6) et Her2/neu (HercepTest), Ki-67 (clone MBI-1).

L'expression des récepteurs hormonaux est évaluée en pourcentage du marquage nucléaire des cellules tumorales et un pourcentage de plus de 10% est considéré comme positif. L'expression du Her2/neu est évaluée selon le protocole du Kit HercepTest, Dako et les résultats sont exprimés en score selon les recommandations de l'ASCO [16]. Les tumeurs sont positives pour les marqueurs cytokératines (CK5/6 et CK8/18) quand on a plus de 5% de cellules marquées. Pour l'EGFR, le seuil de positivité est de 10% de cellules marquées. Les tumeurs avec un ki-67> 14% sont considérées des tumeurs prolifératives.

### Hybridation fluorescente in situ (FISH)

Le protocole Vysis avec le kit PathVysion HER2 DNA Probe (Vysis; Abbott) est utilisé dans l’évaluation d'expression du gène Her2 score 2 selon les recommandations de l'ASCO [16].Une double lecture est effectuée pour chaque lame et les cas considérés amplifiés montrent un ratio HER2/ Chr17 > 2,2

### Analyse statistique

Les données cliniques et biologiques sont traitées en collaboration avec le service d'oncologie médicale. Ces données sont recueillies, codées, saisies sur Microsoft office Excel 2003, puis analysées au moyen du logiciel Epi-Info^’^ Version 3.4. La différence entre les différents groupes est examinée à l'aide du test de Chi-2 ou le test exact de Fisher. Les taux de survie globale (OS) et de survie sans progression (PFS) sont analysés avec la méthode de Kaplan-Meier. Le test log-rank est utilisé pour comparer les différents groupes. La différence statistique est considérée significative lorsque le p ≤ 0,05.

## Résultats

L’étude a porté sur 335 patientes atteintes d'un carcinome mammaire invasif, recrutées au CHU de Fès entre janvier 2007 et septembre 2010. La population de cette région est caractérisée par un âge jeune, avec un âge médian de 45 ans, et des extrêmes allant de 18 à 82 ans. 17% des patientes ont moins de 35 ans (Tableau 1).

**Tableau 1:** Description générale de la population

Caractéristiques	Nombre de cas (%)	
**Age median (ans)**	45	
**Age**	57	17%
≤ 35 ans	57	17%
> 35 ans	278	83%
**Taille tumorale ± SD (cm)**	3,8 +/- 2,6	
**Taille tumorale**		
< 2 cm	87	26%
≥ 2 cm	248	74%
**Grade histologique SBR**		
I	48	14.3%
II	185	55.3%
III	102	30.4%
**Envahissement ganglionnaires (N)**		
N0	93	35,4%
N+	170	64,6%
**Statut métastatique**		
M0	159	73,6%
M1	57	26,4%
ER positive	186	55,5%
PR positive	216	64,5%
Her2 positive	92	27,5%

L’étude clinico-pathologique a montré que le diagnostic du cancer du sein dans cette région est tardif, puisque la taille moyenne des tumeurs est de 3,8±2,6 cm avec 74% des cas qui ont une taille supérieure à 2 cm (T2 et T3). Le nombre des tumeurs grade histologique II et III est élevé (55.3% et 30.4% respectivement) (Tableau 1). Le type histologique prédominant des tumeurs est le carcinome canalaire infiltrant dans 88% des cas, suivi du carcinome lobulaire infiltrant (3,9%), puis le carcinome métaplasique (2,7%) et le carcinome médullaire (2,4%). Les autres types histologiques sont plus rares.

L'envahissement ganglionnaire est déterminé uniquement pour 263 patientes vu que les patientes métastatiques ont bénéficié soit d'une mastectomie de propreté sans curage ganglionnaire soit d'une biopsie.

Le statut métastatique n'est disponible que pour 216 patientes vu le coût et la non disponibilité de la scintigraphie osseuse dans notre formation durant la période d′étude.

64.6% des patientes présentent un envahissement ganglionnaire et 26.4% ont développé des métastases à distance (Tableau 1).

Par ailleurs, l’étude immunohistochimique a révélé que 55.5% des tumeurs sont RE positif, 64.5% sont RP positif et 27.5% sont Her2 positif. A l'issu de ces résultats et selon la nouvelle classification moléculaire, 54.3% des tumeurs sont du groupe luminal A, 16% sont luminal B, 11.3% sont Her2+, 11.3% sont basal-like et 7% sont non-classées (négatives pour tous les biomarqueurs utilisés).

La différence d’âge n'est pas significative chez les patientes des différents groupes moléculaires.

La taille tumorale est supérieure à 3.5 cm (p = 0,43) avec plus de74% des cas qui ont une taille supérieure à 2 cm (T2 et T3) (Tableau 1), cela quelque soit le groupe moléculaire.

En corrélant le type moléculaire des tumeurs avec le grade histologique; le groupe luminal A renferme le plus haut taux du grade histologique I (18.8%) et le plus faible taux du grade histologique III (24.3%), alors que les tumeurs non-classées et basal-like ont un taux élevé de grade histologique III (52.2% et 42% respectivement), une faible proportion de grade I (inférieure à 5.3%) (p = 0,036) et également une faible présence d'emboles vasculaires (26%, p = 0,31)) (Tableau 2). Par ailleurs, il est à noter que l'envahissement ganglionnaire est plus élevé dans les deux groupes moléculaires luminal B et Her2+ (73.9% et 66.7% respectivement); alors qu'il est relativement faible dans le basal-like et les non-classés (55.6% et 60% respectivement, p = 0,59) (Tableau 3). Concernant la diffusion métastatique à distance, il n'y a pas de différence significative entre les groupes moléculaires mis à part le groupe des non-classées où le taux de métastases est élevé (45.5%), alors que le groupe du luminal A renferme la plus faible proportion des métastases (22.9%) (p= 0,67) (Tableau 3).

**Tableau 2:** Prévalence des sous types moléculaires et leurs caractéristiques

Caractéristiques	Tous les Cases	Luminal A	Luminal B	Her2+	Basal-like	non-classée	*P-* value
Nombres des cases (%)	335	182 54,3%	54 16%	38 11,3%	38 11,3%	23 7%	
Age médian		45	44	44	46	43	
Taille tumorale moyenne (cm)		3,6+/-2,5	3,9+/-2,1	3,5+/-2	4,7+/-3	3,7+/-3,7	0,437*
**Statut**							0,510
pré-ménopausé	181 54%	92 50,5%	33 59%	23 60,5%	19 50%	15 65%	
post-ménopausé	154 46%	90 49,5%	22 41%	15 39,5%	19 50%	8 35%	
**Garde SBR**							0,0365
I	48 14,3%	34 18,8%	7 13%	5 13,2%	2 5,3%	0 0%	
II	185 55,3%	104 57%	32 59,2%	18 47,4%	20 52,7%	11 47,8%	
III	102 30,4%	44 24,2%	15 27,8%	15 39,5%	16 42%	12 52,2%	
**Emboles vasculaires**							0,3107
Absence	210 62,7%	113 62,1%	30 55,6%	22 57,9%	28 73,7%	17 73,9%	
Presence	125 37,3%	69 37,9%	24 44,4%	16 42,1%	10 26,3%	6 26,1%	

* test exact de Fisher

**Tableau 3:** Statut ganglionnaire et métastatique dans 4 groupes moléculaires

	Tous les Cases	Luminal A	Luminal B	Her2+	Triple négatifs	*P-* value
**Statut N**						0,593
N0	93 35,4%	53 36,6%	12 26,1%	10 33,3%	18 42,8%	
N+	170 64,6%	92 63,4%	34 73,9%	20 66,7%	24 57,2%	
**Statut métastatique**						0,677
M0	159 73,6%	91 77,1%	27 69,2%	14 73,7%	27 67,5%	
M1	57 26,4%	27 22,9%	12 30,8%	5 26,3%	13 32,5%	

Le traitement et le suivi médical des patientes étaient faits dans le service d'oncologie médical du CHU qui est devenu opérationnel courant l'année 2007. Ainsi, 11% des patientes ont bénéficié d'un traitement chirurgical seul et 28% des cas ont bénéficié d'une chimiothérapie néoadujuvante. La chimiothérapie adjuvante est administrée pour 61% des cas comme suite: Chirurgie associée à une chimiothérapie adjuvante pour 36% des cas, chirurgie associée à une chimiothérapie et radiothérapie adjuvante pour 28% des cas, chirurgie associée à une chimiothérapie-radiothérapie-hormonothérapie adjuvante pour 22% des cas, chirurgie associée à une chimiothérapie-hormonothérapie adjuvante pour 14% des cas. La chimiothérapie utilisée est à base d'anthracyclines (protocole AC60, FEC 100 et /ou taxanes (docetaxel)). Pour l'hormonothérapie, la majorité des patientes ont reçu Tamoxifène et peu de patientes ménopausées ont reçu des anti-aromatases vu le coût de ces derniers.

L'estimation de la survie globale et de la survie sans progression sont réalisées sur réalisées sur 181 patientes pour une période de trois ans. Les résultats obtenus ont montré que 3% des patientes sont décédées et 11% ont récidivé.

**Figure 1:**
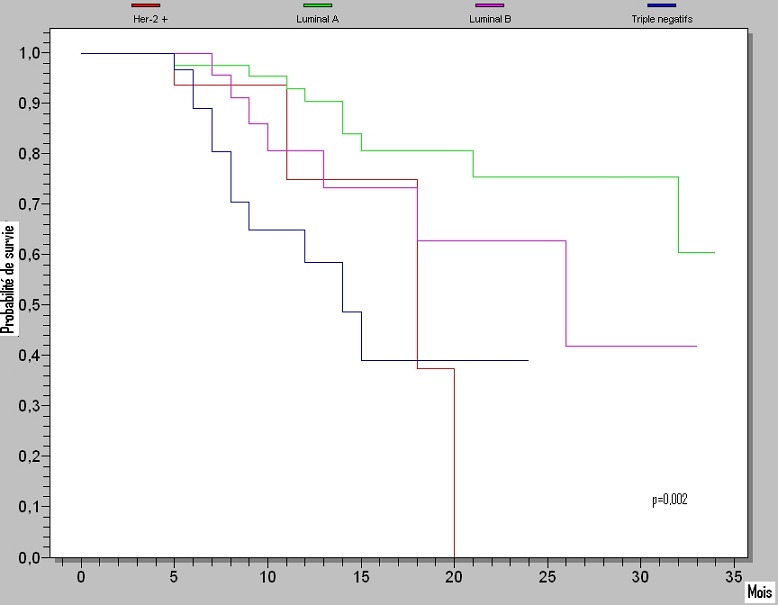
La survie sans progression à 3 ans (p=0,002; test Log-Rank)

**Figure 2:**
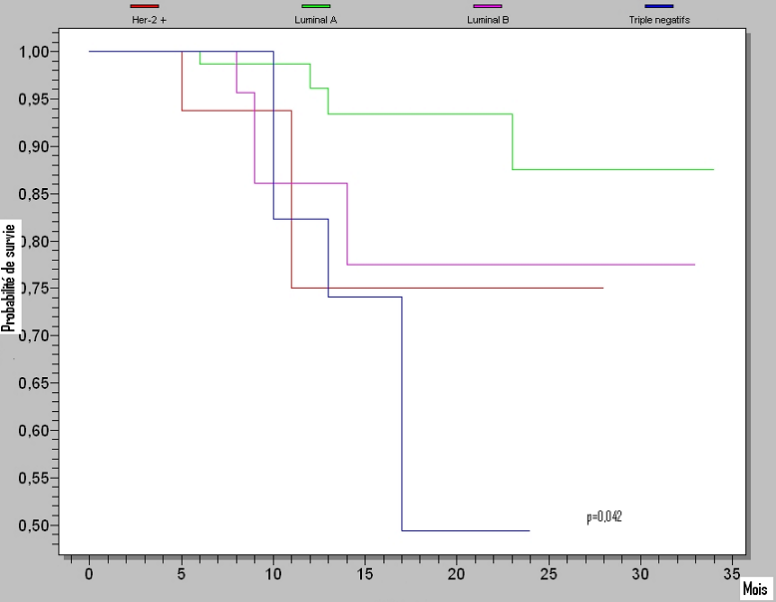
La survie globale à 3 ans (p=0,042; test Log-Rank)

Le taux de survie sans progression à 3 ans diffère significativement (P =0.002) entre le groupe moléculaire luminal A (59%) et les autres groupes: luminal B (41%), Her2+ (38%) et triples négatifs (39%) (Figure 1) par contre le taux de survie globale à 3 ans diffère significativement d'un groupe moléculaire à un autre (p =0.0422); il est de 88% pour le groupe luminal A, 77% pour le groupe luminal B, 75% pour le groupe Her2+ et 49% pour le groupe des triples négatifs (Figure 2).

## Discussion

Le cancer du sein est une maladie complexe et hétérogène, associée à des facteurs cliniques, pathologiques et biologiques largement variables d'une population à une autre [9]. L'expression des récepteurs oestrogéniques (RE), progestéroniques (RP) et le facteur de croissance Her2 doit impérativement être déterminée pour chaque tumeur du sein pour une sélection des patientes en vue d'un bon choix thérapeutique et d'une meilleure prise en charge. Dans cette étude, toutes les tumeurs ont été analysées dans le laboratoire d'anatomie pathologique du CHU Hassan II, sont gradées selon le système SBR et classées en groupes moléculaires après une étude IHC basée sur l'expression des RE, RP, Her2, ki-67, CK5/6, CK8/18 et EGFR. La population étudiée est jeune, avec un âge médian de 45 ans du même ordre que celui retrouvé dans la population jordanienne [17], la taille tumorale moyenne est très élevée (3.8±2.6cm), les grades histologiques II et III sont importants (55.3% et 30.4% respectivement) et le statut ganglionnaire et métastatique est également élevé (46% et 26.4% respectivement). Nous précisons que ces valeurs statistiques sont d'abord discordantes avec ce qui a été publié dans les pays occidentaux [9] et par ailleurs, témoignent d'un diagnostic tardif du cancer du sein au Maroc d'où un dépistage programmé et précoce s'impose pour cette population.

L’étude des profils protéiques par IHC a révélé la présence de cinq groupes moléculaires dont le prédominant est le groupe luminal A (54.3%) ce qui concorde avec la littérature [18–19]. Ce groupe est suivi du luminal B (16%), Her2+ et basal-like avec 11.3% chacun puis le groupe des non-classées (7%). La prévalence des tumeurs basal-like dans cette étude (11.3%) est du même ordre que chez les japonais (8%) [20] alors qu'elle est faible par rapport à ce qui a été rapporté dans l’étude de Carey et al. [9] chez les femmes américaines (20%) et particulièrement chez les afro-américaines (26%). Les tumeurs non-classées représentent 37.7% de l'ensemble des triples négatifs et une prévalence de 7% comme groupe à part. Ces valeurs concordent avec la littérature [9, 20], mais diffèrent de l’étude menée en Arabie Saoudite où la prévalence du groupe des non-classés est de 42.8% [21].

La différence d’âge médian entre les différents groupes moléculaires est non significative, par contre il y a une influence du statut ménopausique sur les groupes Her2+ et luminal B; résultat différent de ce qui a été observé dans l’étude de Carey et al. [9] où les groupes basal-like et luminal A sont influencés par le statut ménopausique. La prévalence des groupes moléculaires est donc vraisemblablement liée à l'origine de la patiente et au statut ménopausique ce qui confirme également les travaux publiés antérieurement [9, 20, 21].

Dans cette étude, la taille tumorale est élevée quelque soit le groupe moléculaire, et ce facteur clinique conditionnerait la survenue de métastases et le taux de survie globale des patientes [22]. Ainsi, l’étude de Tabar et al [23] a montré que le taux de survie à 10 ans variait en fonction de la taille tumorale, il était de 65 et de 50% pour des tumeurs T2 et T3 respectivement. Par ailleurs, le groupe moléculaire luminal A, dans cette étude, s'associe avec la plus faible proportion de grade histologique III et d'emboles vasculaires, cela concorde avec les données de la littérature [9, 18], et ce groupe est considéré de bon pronostic pour la population étudiée. Ainsi, le taux de survie globale à 3 ans des patientes étudiées de ce groupe est le plus élevé (88%). Les autres groupes moléculaires présentent des particularités différentes les uns des autres: le groupe luminal B est corrélé à un pourcentage élevé d'emboles vasculaires (44.4%) et d'envahissement ganglionnaire (73.9%), puis le groupe Her2+ s'associe à un taux élevé de grade histologique III (39.5%), d'emboles vasculaires (42.1%) et d'envahissement ganglionnaire (66.7%). Les tumeurs non-classées sont particulièrement agressives avec un taux élevé de grade histologique III (52.2%) et d'envahissement ganglionnaire (60%). Cependant, les tumeurs basal-like ne se retrouvent corrélées qu'avec une proportion relativement élevée de grade histologique III (42%). Ces résultats montrent une corrélation étroite entre les groupes moléculaires luminal B et Her2+ avec l'envahissement ganglionnaire ce qui n'est pas le cas avec le groupe basal-like, cela est concordant avec les travaux de Carey et al. [9].

Etant donné que l'effectif des tumeurs non-classées dans la population étudiée est très faible (12 patientes) par rapport aux autres groupes moléculaires, il est intéressant de regrouper les tumeurs basal-like et les tumeurs non-classées en un seul groupe «triples négatifs » pour une meilleure interprétation des résultats. Ainsi, le groupe des triples négatifs se caractérise par une taille tumorale élevée (4.45 cm), une proportion élevée de grade histologique III (46%) et également une fréquence importante de métastases viscérales ce qui explique la fréquence relativement basse d'envahissement ganglionnaire par rapport aux autres groupes moléculaires. Durant une période de 3 ans, le taux de survie globale des patientes de ce groupe est significativement plus bas que celui des patientes du groupe luminal A (p =0.002).

Les tumeurs du groupe luminal B dans notre contexte sembleraient avoir un pronostic plus péjoratif que les tumeurs Her2+. En effet, la taille tumorale est légèrement plus importante dans le luminal B (3.9 versus 3.5 cm), le pourcentage des emboles vasculaires est sensiblement plus élevé (44.4% versus 42.1%), l'envahissement ganglionnaire est augmenté (73.9% versus 66.7%) et la diffusion métastatique à distance est plus importante (30.8% versus 26.3%). Cependant, les données de la survie globale à 3 ans n'ont pas révélé de différence significative entre ces deux groupes moléculaires, ceci pourrait être biaisé par l'effectif peu important des patientes étudiées et par le faible nombre de mois du suivi médical.

La rechute métastatique dans les différents groupes moléculaires ne peut être évaluée que si on connait la durée de sa survenue, ce qui est très rare puisque plus de 70% des patientes de cette région consultent avec une taille tumorale élevée (supérieure à 2 cm) associée à un envahissement ganglionnaire et parfois à des métastases à distance ce qui témoignent d'un stade avancé des tumeurs.

## Conclusion

La détermination des groupes moléculaires est actuellement indispensable pour une meilleure stratégie thérapeutique des patientes selon le profil génique ou protéique des tumeurs. L'IHC peut servir de surrogate au microarray pour définir les sous types moléculaires. Le groupe luminal B est différent du groupe luminal A et de pronostic péjoratif vis-à-vis du groupe Her2+. L'agressivité de ces deux groupes moléculaires sera réalisée en estimant la durée de survie sur un minimum de cinq ans et la survie globale sur un effectif de patientes plus important, de même il serait intéressant d’étudier l'expression génique des deux groupes luminaux. Par ailleurs, le groupe des triples négatifs est d'agressivité significativement élevée par rapport aux autres groupes moléculaires. Dans cette étude, il faudrait prendre également en considération la taille tumorale, l’âge, le grade histologique et le profil moléculaire puisqu'ils concordent avec les facteurs pronostiques de rechute locorégionale après traitement chirurgical.
